# Perceptions about the dialysis modality decision process among peritoneal dialysis and in-center hemodialysis patients

**DOI:** 10.1186/s12882-018-1096-x

**Published:** 2018-10-29

**Authors:** Jarcy Zee, Junhui Zhao, Lalita Subramanian, Erica Perry, Nicole Bryant, Margie McCall, Yanko Restovic, Delma Torres, Bruce M. Robinson, Ronald L. Pisoni, Francesca Tentori

**Affiliations:** 1 Arbor Research Collaborative for Health, 340 E. Huron Street Suite 300, Ann Arbor, MI 48104 USA; 2 University of Michigan Health System, Ann Arbor, MI USA; 3 Advisory panel, Ann Arbor, MI USA; 4 Vanderbilt University Medical Center, Nashville, TN USA

**Keywords:** End-stage renal disease, Dialysis modality, Hemodialysis, Peritoneal dialysis, Renal replacement therapy

## Abstract

**Background:**

Patients reaching end-stage renal disease must make a difficult decision regarding renal replacement therapy (RRT) options. Because the choice between dialysis modalities should include patient preferences, it is critical that patients are engaged in the dialysis modality decision. As part of the Empowering Patients on Choices for RRT (EPOCH-RRT) study, we assessed dialysis patients’ perceptions of their dialysis modality decision-making process and the impact of their chosen modality on their lives.

**Methods:**

A 39-question survey was developed in collaboration with a multi-stakeholder advisory panel to assess perceptions of patients on either peritoneal dialysis (PD) or in-center hemodialysis (HD). The survey was disseminated to participants in the large US cohorts of the Dialysis Outcomes and Practice Patterns Study (DOPPS) and the Peritoneal DOPPS (PDOPPS). Survey responses were compared between PD and in-center HD patients using descriptive statistics, adjusted logistic generalized estimating equation models, and linear mixed regression models.

**Results:**

Six hundred fourteen PD and 1346 in-center HD participants responded. Compared with in-center HD participants, PD participants more frequently reported that they were engaged in the decision-making process, were provided enough information, understood differences between dialysis modalities, and felt satisfied with their modality choice. PD participants also reported more frequently than in-center HD participants that partners or spouses (79% vs. 70%), physician assistants (80% vs. 66%), and nursing staff (78% vs. 60%) had at least some involvement in the dialysis modality decision. Over 35% of PD and in-center HD participants did not know another dialysis patient at the time of their modality decision and over 60% did not know the disadvantages of their modality type. Participants using either dialysis modality perceived a moderate to high impact of dialysis on their lives.

**Conclusions:**

PD participants were more engaged in the modality decision process compared to in-center HD participants. For both modalities, there is room for improvement in patient education and other support for patients choosing a dialysis modality.

**Electronic supplementary material:**

The online version of this article (10.1186/s12882-018-1096-x) contains supplementary material, which is available to authorized users.

## Background

Over 120,000 patients reaching end-stage renal disease (ESRD) every year in the United States (US) are faced with a complex and difficult decision regarding renal replacement therapy (RRT) modality options [1]. Although kidney transplant results in the best clinical outcomes, 97% of ESRD patients will require dialysis, most frequently peritoneal dialysis (PD) or in-center hemodialysis (HD) [1]. Although clinical contraindications restrict modality choice in occasional cases, most patients are candidates for both PD and HD [2]. Either dialysis modality may be a better fit for a specific patient based on dialysis treatment characteristics and their impact on daily life. Thus, the choice between modalities should center on patient preferences, and it is critical that patients are included and engaged in the dialysis modality decision [3, 4]. This is supported by increasing evidence that aligning treatment with patient preferences may improve adherence to therapy, quality of life, and ultimately better medical outcomes [5–8].

Clinical practice guidelines support the role of patients and their caregivers in the dialysis modality decision-making process [5, 9–11]. Unfortunately, interviews with dialysis patients show that many did not feel they were given an active choice of modality [3, 12–14], despite wanting to be involved in decision-making [3, 15]. To do so effectively, patients and their caregivers must have a comprehensive understanding of the differences between dialysis modalities and their impacts on daily life [16, 17]. However, previous studies have shown many patients felt unprepared and ill-informed about starting dialysis and about different dialysis modalities [13, 18]. Dialysis education can prepare patients for shared decision-making, and may ultimately lead to better outcomes through more active engagement in care [16, 19–22].

The Empowering Patients on Choices for Renal Replacement Therapy (EPOCH-RRT) study, supported by the Patient-Centered Outcomes Research Institute (PCORI), sought to develop a decision aid (http://choosingdialysis.org) to help patients choose a dialysis modality. To determine factors that are most important to patients when considering dialysis, the EPOCH-RRT study first conducted semi-structured interviews of patients with chronic kidney disease (CKD) and patients undergoing PD or in-center HD. Some of the factors participants identified were independence, flexibility in daily lives, concerns about looks, and quality and quantity of life [14]. For this part of the EPOCH-RRT study, we developed a survey partly based on the information gained from interviews, and we administered the survey to the large, national US samples of participants in the Dialysis Outcomes and Practice Patterns Study (DOPPS) and the Peritoneal DOPPS (PDOPPS). Our aim was to assess participants’ perceptions of the dialysis modality decision-making process and compare the impact of their chosen modality on their lives with the goal to inform efforts to increase patient engagement and ultimately contribute to improving patient-centered outcomes.

## Methods

### Survey design

A 39-question survey (Additional file 1: Figure S1) to assess participants’ experiences with the dialysis modality decision and factors that participants had previously identified as important (“patient-centered outcomes”) was developed in collaboration with a multi-stakeholder advisory panel and partly based on the analysis of qualitative data collected from 180 advanced CKD participants [14]. The advisory panel included dialysis patients, caregivers (e.g., dialysis patients’ family members), and patient advocates (e.g., social workers), who provided perspectives on experiences with CKD and ESRD. The advisory panel informed the development of the survey, tested the survey for readability and comprehension, and helped to review and finalize survey questions. Given the panel members’ expertise with the target study population and their personal experience as either patients or caregivers, they were able to assess face validity, interpretability, relevance, and comprehensiveness of questionnaire items. Questionnaires for PD vs. in-center HD patients were identical except for the exchange of the words “peritoneal dialysis” vs. “hemodialysis.” The survey was designed for both paper and electronic (tablet) formats, with substantial advisory panel input on layout and design of the electronic survey. The survey was professionally translated from English to Spanish and reviewed by Spanish-speaking members of the institutional review boards, then made available to study participants in either English or Spanish.

Participants were asked whether they were told they had a choice between PD and HD when starting dialysis and to indicate if their involvement in this decision was more, less, or just what they wanted. The survey proceeded with three sets of questions: (1) Participants ranked the degree to which 10 groups of family members, peers, and clinical staff were involved in their dialysis modality decision, as suggested by the Decisional Needs Assessment in Populations [23]. A Cronbach’s alpha estimate of 0.89 indicated good reliability of these questions to measure involvement of others in the decision process. (2) Participants rated their level of agreement with nine statements focused on their recollection of their experiences and satisfaction with their dialysis modality decision. This set of questions was adapted for the current study population from the COMRADE scale [24] and Decision Regret Scale [25] and had a Cronbach’s alpha estimate of 0.86. Additionally, participants were asked whether the information they had received before starting dialysis was more, less, or just the amount that they had wanted and if they and their doctor had agreed on the type of dialysis that was best for them. (3) Participants ranked the degree to which dialysis affected 16 factors compared with before starting dialysis. These factors were chosen directly based on previous research of the themes most often reported as important to patients when choosing a dialysis modality [14]. Cronbach’s alpha for this set of items was 0.93.

### Participant enrollment

**Fig. 1:**
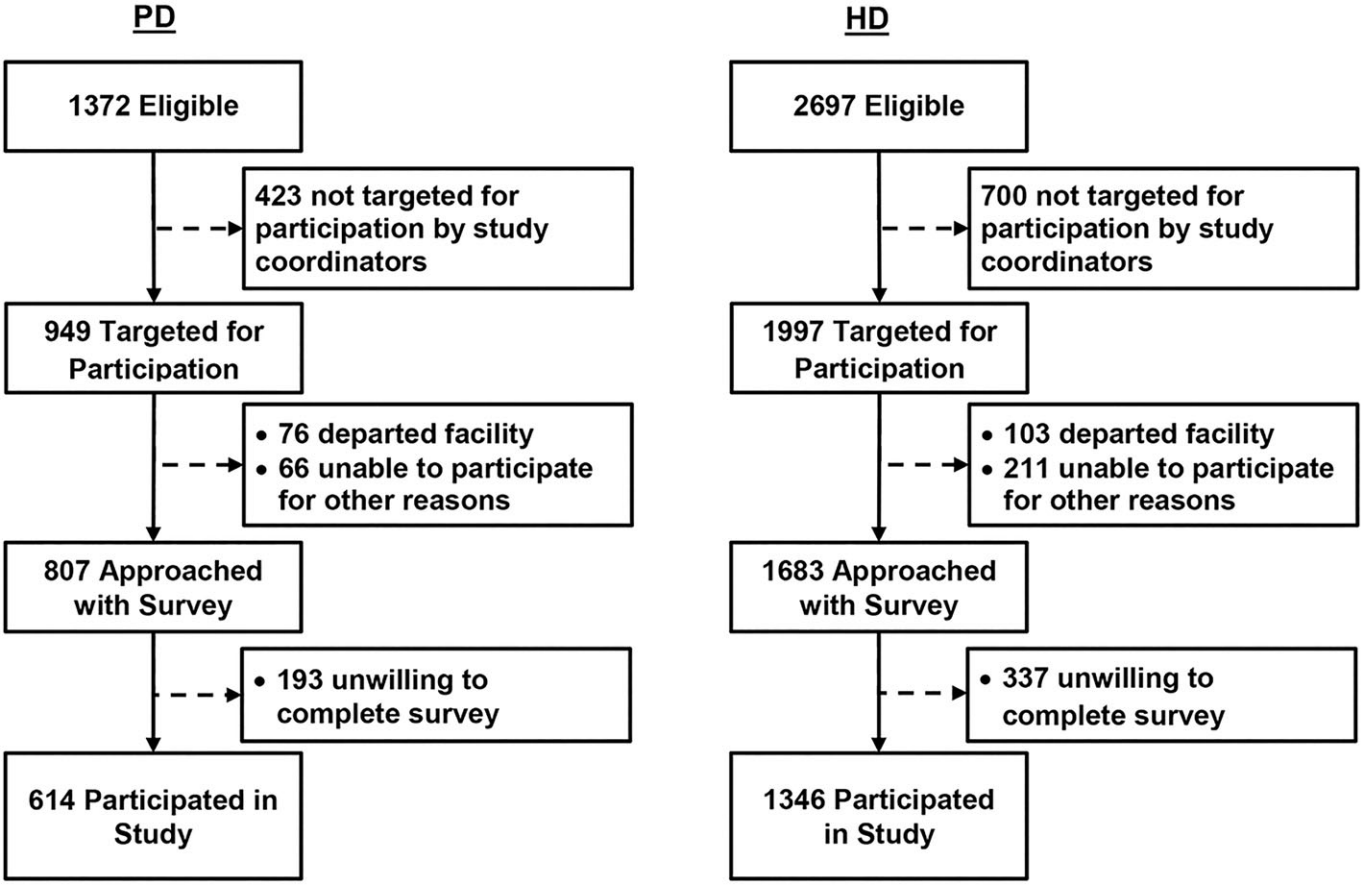
Recruitment of Study Participants

The DOPPS and PDOPPS are ongoing, international prospective cohort studies of dialysis facility practices and patient outcomes for adult in-center HD and PD participants, respectively [26–28]. DOPPS and PDOPPS participants are selected randomly from a national sample of dialysis facilities. All consented participants in the US DOPPS and PDOPPS studies were eligible for the EPOCH-RRT study. Study coordinators targeted as many eligible participants as possible between February 2015 and August 2015 to participate in the EPOCH-RRT survey (Fig. 1). Some participants departed the dialysis facility before study coordinators could approach them with the survey or were unable to participate due to other reasons, such as cognitive, physical, language, or social impediments. Others were approached for participation but unwilling to complete the EPOCH-RRT survey. Facilities were randomly assigned to receive the survey on either paper or tablet platforms. Local institutional review boards (Ethical and Independent Review Services #13016, Henry Ford Health Systems #8144, University of Michigan HUM00073058) approved all study procedures.

### Statistical analysis

For questions on the level of involvement of clinical staff, families, and peers in the dialysis modality selection, we treated the responses as continuous outcomes. Each degree of involvement (i.e., not at all, somewhat, moderately, very much, or extremely) was assigned an integer value from 1 to 5 such that the difference between two adjacent levels represented a 1-unit change. For outcomes on experiences and satisfaction with the dialysis modality decision, responses were dichotomized into agreement (agree or strongly agree) vs. non-agreement (strongly disagree, disagree, or neither agree or disagree) for better model fit and ease of interpretation. For outcomes on factors important to patients, responses were also dichotomized into a large impact (very much or extremely) vs. not large impact (not at all, somewhat, or moderately). Participants who reported not applicable were excluded from analyses of each corresponding question. Missing responses for each question were excluded.

For dichotomized outcomes (experiences and satisfaction with the dialysis modality decision and factors important to patients), logistic generalized estimating equation (GEE) regression models were used to compare outcomes between PD and in-center HD participants. An exchangeable working correlation matrix was used to account for participant clustering within facility. For continuous outcomes (involvement of clinical staff, families, and peers), linear mixed regression models were used to compare dialysis modality, accounting for clustering by including a random intercept for each facility. The primary predictor in all models was dialysis modality, and all models were adjusted for age, sex, black race, time on dialysis (vintage), and diabetes. Adjusted differences in probabilities or levels of each outcome between PD and in-center HD, along with 95% confidence intervals (CI), were estimated using model parameter estimates. Predicted probabilities from logistic regression models were estimated using means for continuous adjustment covariates and most frequent categories for categorical adjustment covariates.

We tested for an interaction between modality and time on dialysis (< 1 year vs. ≥1 year and < 3 years vs. ≥3 years) in each model to assess whether dialysis vintage modified differences in modality. Similarly, among the subgroup of patients with such information, we tested for an interaction between modality and having prior RRT experience (i.e., in-center HD patients with prior PD experience, PD patients with prior in-center HD experience, and in-center HD or PD patients with prior transplant). Because each interaction analysis involved 38 different hypothesis tests, we applied the Benjamini-Hochberg correction for multiple comparisons to control for false discovery rate [29]. We also conducted two sets of sensitivity analyses: 1) we added paper or tablet platform as an additional adjustment factor and tested for dialysis modality effect modification by platform; 2) for dichotomized outcomes, we treated them as continuous variables using linear models and treated them as ordinal using proportional odds models. All analyses were conducted using SAS, Version 9.4 (SAS Institute Inc., 2013, Cary, NC) or Stata, Version 13.1 (StataCorp, 2013, College Station, TX).

## Results

### Study participants

Out of 807 PD and 1683 in-center HD participants approached for participation, 614 (76.1%) PD participants from 55 facilities and 1346 (80.0%) HD participants from 80 facilities responded to at least one question in the survey (Fig. 1). Participant characteristics are shown in Table 1. Compared to in-center HD participants, PD participants were younger and were less likely to be black, on average. PD participants also had shorter dialysis vintage, with 46% having started dialysis less than 2 years ago compared to 32% for in-center HD participants. Age, sex, and race distributions of our study sample were similar to those of the US dialysis population, while participants in our study had shorter time on dialysis, on average, than point prevalent dialysis patients in the US in 2013 when these data were collected [1].

**Table 1:** Patient characteristics, by dialysis modality

Variable	PD (*n* = 614)^a^	HD (*n* = 1346)^a^
Patient age, mean (SD) years	59.9 (15.0)	63.0 (14.5)
Male	53.9%	57.4%
Race		
White	70.2%	59.5%
Black	23.0%	35.8%
Other	6.8%	4.7%
Time on dialysis		
0 to < 6 months	5.6%	6.2%
6 to < 12 months	11.4%	8.6%
12 to < 36 months	46.1%	31.1%
36+ months	30.6%	54.1%
Diabetes	41%	43.3%

^a^One PD patient and 9 HD patients were missing demographic data

### Survey completion

Out of 39 total questions, the median (interquartile range) number of questions answered was 36 (33–38) among PD participants and 35 (32–37) among in-center HD participants. The amount of missingness for each question ranged from 3 to 7%, with the exception of question two, regarding the amount of patient involvement in the dialysis modality decision compared to what the participant wanted. This question was left unanswered by 11% of PD participants and 36% of in-center HD participants. In addition, there was a technical error with the question, “I know the disadvantages of hemodialysis compared to peritoneal dialysis” on some of the tablet questionnaires disseminated to in-center HD participants. Therefore, responses to these questions among tablet users (43%) were suppressed and only responses from paper users were used for analyses.

### Survey implementation platform

Among participants < 65 years old, response rates across platform (i.e., paper vs. tablet) were similar among both PD (63.2% paper vs. 66.1% tablet) and in-center HD (68.4% paper and 71.9% tablet) participants. However, participants older than 65 who were offered tablets had lower response rates than those offered paper surveys for PD (72.5% paper vs. 55.7% tablet) and in-center HD (69.4% paper vs. 59.7% tablet).

### Experience regarding dialysis modality choice

PD participants reported that they were more frequently (93%) told that they had a choice between dialysis modalities than were in-center HD participants (66%). 10% of PD participants and 20% of HD participants felt their involvement in the type of dialysis they would start on was either more than or less than they wanted, rather than just what they wanted.

### Involvement of clinical staff, family, and peers

**Fig. 2:**
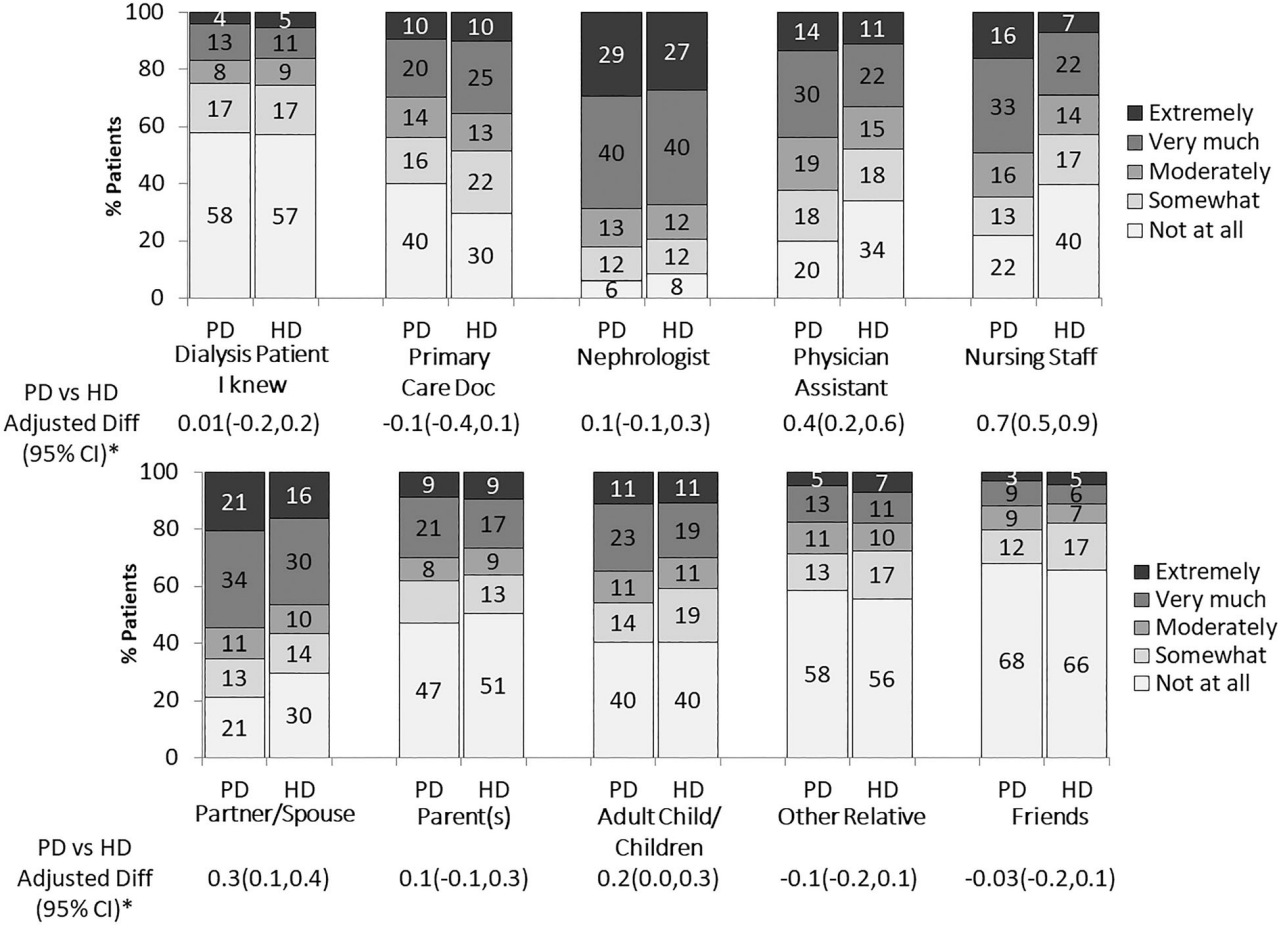
Involvement of family and peers in the dialysis modality decision. Patients who reported not applicable (range: 3% for nephrologist to 35% for peer and 47% for adult child/children) were excluded from the relevant question. *Adjusted differences in the degree of involvement of family members and peers between PD and in-center HD patients. Estimates from linear mixed regression models adjusted for age, sex, black race, time on dialysis, and diabetes, and accounting for facility clustering

Clinical staff members, especially nephrologists, were most frequently involved in the dialysis modality decision overall (Fig. 2). Compared to in-center HD, fewer PD participants reported involvement of primary care doctors (60% vs. 70%). Greater differences were observed in the two modalities when it came to involvement of other clinical staff, with for example, 22% of PD participants and 40% of in-center HD participants reporting no involvement at all of nursing staff in the dialysis decision. In adjusted models, the mean [95% CI] level of involvement of physician assistants was 0.4 [0.2,0.6] higher for PD patients and of nursing staff was 0.7 [0.5,0.9] higher for PD patients compared to in-center HD patients. Less than 65% of all participants reported knowing someone on dialysis at the time of their modality decision; among them, over 50% recalled no peer involvement. Also, more PD participants than in-center HD participants reported at least some involvement of partners/spouses (PD 79%, with 55% reporting very much or extremely; in-center HD 70%, with 46% reporting very much or extremely; adjusted mean [95% CI] difference of 0.3 [0.1,0.4] between PD and HD). For both PD and in-center HD participants, involvement of other family and friends was low to moderate (32–60%).

### Experiences and satisfaction with dialysis modality decision

**Fig. 3:**
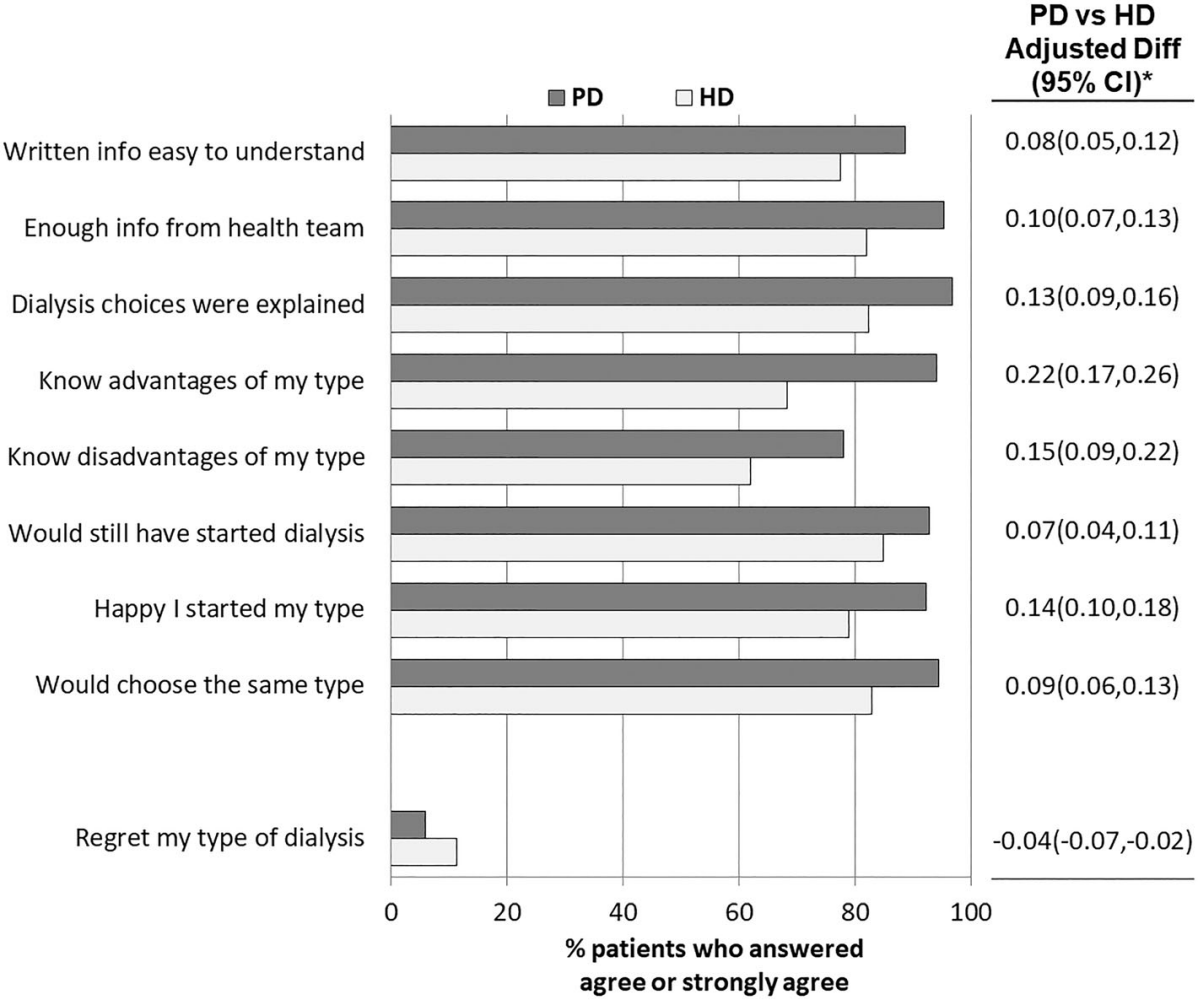
Patients’ self-reported experience and satisfaction with the dialysis modality decision. *Difference in probability and 95% confidence interval (CI) of agreement with each statement comparing PD vs. in-center HD. Estimates from logistic GEE model adjusted for age, sex, black race, time on dialysis, and diabetes, and accounting for facility clustering. Adjusted differences in predicted probabilities were calculated for a white, non-diabetic male of average age and average vintage

In-center HD participants felt less informed and less confident than PD participants at the time of the dialysis modality decision and were less satisfied with their modality choice (Fig. 3). PD participants more often felt the information they were given was enough and easy to understand, with adjusted differences [95% CI] between PD and HD in the probability of agreement of 0.10 [0.07,0.13] and 0.08 [0.05,0.12], respectively. PD participants more frequently agreed that dialysis choices were explained (0.13 [0.09,0.16]), they understood the advantages (0.22 [0.17,0.26]) and disadvantages (0.15 [0.09,0.22]) of their dialysis modality type, and they were happy with their type of dialysis (0.14 [0.10,0.18]). Almost all PD participants felt their dialysis choices had been explained in a way that was easily understandable, whereas close to 20% of in-center HD participants did not. Only 6% of PD participants regretted their choice of dialysis modality, compared with 11% of in-center HD participants (adjusted difference between PD and HD of − 0.04 [− 0.07,-0.02]). While 26% of PD participants reported that the information they had before starting dialysis was either more (9%) or less (17%) than they wanted, rather than just what they wanted, 36% of in-center HD participants reported they had either more or less information (11% and 25%, respectively) (*p* = 0.178). 95% of PD participants and 84% of in-center HD participants reported that they and their doctor agreed about the type of dialysis that was best for them (*p* < 0.05).

### Impact of dialysis on patients’ lives

**Fig. 4:**
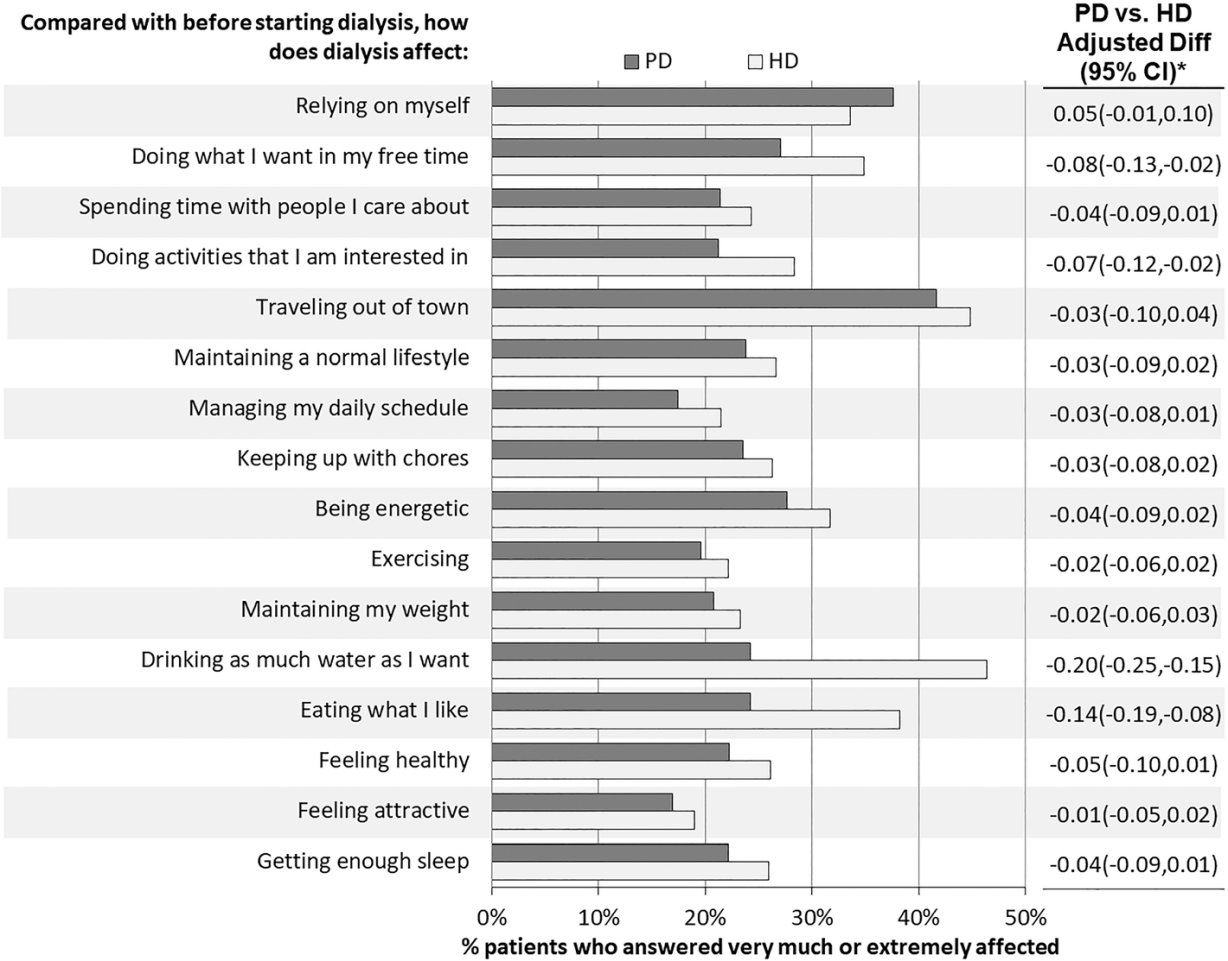
Effect of dialysis on patient-centered outcomes. Patients who reported not applicable (range: 1% to 9%) were excluded from the relevant question. * Difference in probability and 95% confidence interval (CI) of a large impact of dialysis on each factor comparing PD vs. in-center HD. Estimates from logistic GEE model adjusted for age, sex, black race, time on dialysis, and diabetes, and accounting for facility clustering. Adjusted differences in predicted probabilities were calculated for a white, non-diabetic male of average age and average vintage

A sizable number of participants on both in-center HD and PD reported that dialysis had a large impact on all factors assessed (range 17–46%, Fig. 4). In-center HD participants were more affected than PD participants for 15 of 16 factors, but the differences were generally small. PD participants felt their dialysis modality affected reliance on themselves slightly more than HD participants, with an adjusted difference in probability of agreement of 0.05 [− 0.01,0.10] between PD and HD. The largest differences between PD and in-center HD participants were observed for the factors: doing what I want in my free time (− 0.08 [− 0.13,−0.02]), doing activities I am interested in (hobbies) (− 0.07 [− 0.12,−0.02]), drinking as much water as I want (− 0.20 [− 0.25,−0.15]), and eating what I like (− 0.14 [− 0.19,−0.08]).

### Effect modification and sensitivity analyses

The interaction between modality and time on dialysis (using 1 year or 3 years as a cut-point) was not statistically significant in any model. The interaction between modality and prior RRT experience was also not statistically significant in any model among the *N* = 140 patients with prior RRT experience and *N* = 1159 patients without prior RRT experience. Therefore, we did not find evidence that time on dialysis or prior RRT experience modified the differences in outcomes between PD and in-center HD. For all outcomes, similar results were obtained after adjusting additionally for platform (tablet vs. paper). In analyses testing for interactions between modality (PD vs. in-center HD) and questionnaire platform (tablet vs. paper), we found little effect modification. In sensitivity analyses treating dichotomized outcomes as continuous or ordinal, results were similar but model assumptions (i.e., normality or proportional odds) were sometimes violated. Thus, we concluded that logistic regressions were most appropriate for these outcomes.

## Discussion

By collaborating with an advisory panel and using analyses from qualitative data collected from EPOCH-RRT participant interviews, we developed a survey specifically designed to focus on patient-centered outcomes. This approach was consistent with PCORI goals for multi-stakeholder engagement in research and was invaluable for informing the survey content and interpretation of results. Our survey results showed that that participants who were on PD were more informed and engaged in dialysis modality decision-making compared with in-center HD participants overall. This may be expected, given that PD participants undergo intense training coordinated by clinical staff and that this dialysis technique has an impact on an entire household’s quality of life [30]. Therefore, those who choose PD may already be more involved in their own care and likely more receptive to education they receive. Nonetheless, the low involvement of several groups in the dialysis modality decision for both PD and in-center HD participants demonstrates an opportunity to increase family and peer engagement to promote shared decision-making. Such engagement may result in a better fit of the dialysis modality with each patient’s life, as well as improved experience for their families and other caregivers [31–33]. The large number of dialysis participants who did not know someone else on dialysis highlights a potentially useful but underutilized resource, for example. Beyond having support of peers, peer mentoring programs have been successful in different clinical conditions [34–37], and anecdotal evidence indicates that existing peer support programs in dialysis are highly valued by patients and their caregivers [38, 39]. By improving awareness of and access to peers and peer mentors, patients new to dialysis may benefit from increased practical information about dialysis, empathy and understanding, advice on coping strategies, and a greater sense of empowerment and agency [39].

PD participants were much more likely than in-center HD participants to report greater involvement in the dialysis modality decision. Previous studies have found that deficiencies in awareness of options are a barrier to choosing PD and that educational interventions can increase PD use [40–42]. Thus, health care providers may offer PD as an option more often to patients with higher health literacy or better self-care abilities. PD participants also more frequently indicated they were happy with the modality they chose compared to in-center HD participants. This result may reflect a more deliberate and informed decision-making process among PD participants and/or greater involvement in the dialysis modality decision. It may also reflect a more positive perception of the dialysis experience specifically due to self-reinforcement from feeling involved in the modality decision process. Still, many PD participants did not know the disadvantages of their modality and did not feel they had written information that was easy to understand. In both PD and HD groups, such a lack of information and regret in the dialysis modality choice points to opportunities to improve ESRD education for advanced CKD patients. Increased education could then lead to increased understanding of dialysis modalities and satisfaction with treatment, especially among those who ultimately choose in-center HD [19, 20].

Several impactful factors were more frequently identified by in-center HD participants compared to PD participants. Some of these differences may be explained not only by the differences in modality technique but also the location and medical environment where dialysis is performed. For example, clinical characteristics (e.g., lack of residual urine output) of HD patients may require more restrictive diets and fluid intake, while technical aspects of in-center HD (e.g., intermittent dialysis in a facility setting) often limit the time in-center HD patients have for their own interests like travel [43]. Some in-center HD patients have also reported that dialyzing in a clinical setting and being surrounded by other patients makes them feel less healthy, although this opportunity to interact with other patients in the in-center setting was not always perceived as a negative aspect of in-center HD [3].

Overall, the proportion of participants who skipped each question was low, supporting the fact that the survey questions were appropriate and easily interpretable by most dialysis participants. This likely reflected the high engagement of the advisory panel in the development of the survey and reviews of survey questions. There was a higher amount of missingness for one question about the amount of the participant’s involvement in the dialysis modality decision. The reasons for which participants did not answer this question could include not having preconceived desires about involvement in the dialysis modality decision and/or unwillingness to admit low involvement. Both suggest that more effort should be made to give participants adequate choice and involvement in their dialysis modality decision process and to monitor patient involvement during that process [44].

There are a few limitations of our study worth noting. First, survey questions asked the extent to which participants felt affected by dialysis, but did not ask whether participants perceived the effects to be positive or negative. Therefore, the direction of impact can only be speculated based on what is known about the different modalities until it can be elucidated by future research. Second, patients’ perceptions of others’ involvement in PD training may have inflated their perceptions of involvement in the dialysis modality decision process. While it is plausible that those who must later be involved in training also have some involvement in the decision process, we cannot separate the two periods of involvement in our data. Third, surveys were administered to both incident and prevalent dialysis participants, so the time between dialysis initiation and survey was variable and experiences reflect those of survivors. Particularly for participants who had longer dialysis vintage, recall bias may have affected survey responses related to the dialysis modality decision. However, we have no reason to believe that the recall bias would be different across PD and in-center HD participants, indicating that comparisons between modalities may still have little bias. We also adjusted for vintage in models and found no evidence of modality effect modification by vintage. Fourth, we did not have information on whether participants in the study had contraindications to either dialysis modality, which also may have affected survey responses. For example, some in-center HD participants may not have had PD available if starting dialysis acutely (information not available in our data), which limited their exposure to PD information. Still, these patients should be empowered with information to decide whether to stay on in-center HD or switch to PD when available. Finally, comparisons between PD and in-center HD participants may have been confounded by factors like frailty, comorbid diseases, educational background, social support, and dialysis modality history. Although these variables were not available for analysis, we did control for the most common comorbidity of diabetes and age which is likely to be correlated with frailty.

## Conclusions

Our study has several strengths and important implications for ESRD patients and their families as well as for health care providers. By comparing the experiences of PD and in-center HD patients, we identified important differences between these modalities. We found several aspects of the dialysis modality decision that require improvement for both PD and in-center HD patients, including patient education, access to peers, and other support. Increased efforts are needed to encourage a holistic approach to care that involves multiple stakeholders and provides resources such as decision aids for patients facing the choice between dialysis modalities. It is our hope that a focus on education and multi-stakeholder engagement in the dialysis modality decision will empower patients, their caregivers, and health care professionals to collaboratively choose a dialysis modality that best fits the individual patient, with the goals of ultimately improving satisfaction and health outcomes.

## Additional file

Additional file 1: **Figure S1.** Survey for PD Patients. HD patient survey was identical, except with the exchange of the words “peritoneal dialysis” and “hemodialysis.” (DOCX 447 kb)

## Abbreviations

CKD: Chronic kidney disease
DOPPS: Dialysis Outcomes and Practice Patterns Study
EPOCH-RRT: Empowering Patients on Choices for RRT
ESRD: End-stage renal disease
GEE: Generalized estimating equation
HD: Hemodialysis
PCORI: Patient-Centered Outcomes Research Institute
PD: Peritoneal dialysis
PDOPPS: Peritoneal DOPPS
RRT: Renal replacement therapy
