# Supplementary material for: Perceptions about the dialysis modality decision process among peritoneal dialysis and in-center hemodialysis patients
Source: BMC Nephrol. 2018 Oct 29;19:298. doi: 10.1186/s12882-018-1096-x (PMC6206892; doi:10.1186/s12882-018-1096-x)
Supplement: Supplementary file 1 — Figure S1. Survey for PD Patients. HD patient survey was identical, except with the exchange of the words “peritoneal dialysis” and “hemodialysis.” (DOCX 447 kb) [file 12882_2018_1096_MOESM1_ESM.docx]

# Perceptions about the Dialysis Modality Decision Process among Peritoneal Dialysis and In-Center Hemodialysis Patients

Jarcy Zee, PhD,^1^ Junhui Zhao, PhD,^1^ Lalita Subramanian, PhD,^1^ Nicole Bryant,^2^ Margie McCall, BA,^2^ Yanko Restovic,^2^ Delma Torres,^2^ Bruce Robinson, MD,^1,3^ Ron Pisoni, PhD^1^, Francesca Tentori, MD^1,4^.

^1^Arbor Research Collaborative for Health, Ann Arbor, MI; ^2^ Advisory panel, Ann Arbor, MI; ^3^ University of Michigan, Department of Internal Medicine, Ann Arbor, MI, USA ^4^Vanderbilt University Medical Center, Nashville, TN.

**Supplemental material**

**Figure S1: Survey for PD Patients.** HD patient survey was identical, except with the exchange of the words “peritoneal dialysis” and “hemodialysis.”****
